# Supplementary material for: Repurposing the HMG-CoA reductase inhibitor atorvastatin for SRD5A3-congenital disorder of glycosylation
Source: Commun Med (Lond). 2026 Jul 29;6:517. doi: 10.1038/s43856-026-01791-4 (PMC13642943; doi:10.1038/s43856-026-01791-4)
Supplement: Supplementary file 1 [file 43856_2026_1791_MOESM1_ESM.pdf]

nature portfolio

Peer Review File

Repurposing the HMG-CoA Reductase Inhibitor Atorvastatin for SRD5A3-Congenital Disorder of Glycosylation

Corresponding Author: Dr Kristin Kantautas

This file contains all reviewer reports in order by version, followed by all author rebuttals in order by version.

Version 0:

Reviewer comments:

Reviewer #1

(Remarks to the Author)
This manuscript describes the generation of a pre-clinical c.elegans model that carries a pathogenic nonsense variant in the human ortholog of SRD5A3. They show homozygous mutant worms and knockout worms have phenotypic and biochemical similarities to humans with biallelic pathogenic variants in SRD5A3, which is associated with SRD5A3-related congenital disorder of glycosylation. The authors performed a drug screen to identify small molecules that can rescue the phenotypes observed in the mutant worms. Among the 75 molecules that showed phenotypic rescue, they focused primarily on atorvastatin, an FDA-approved HMG-CoA reductase inhibitor. Biochemically, they showed this drug partially restored polyprenol-to-dolichol ratios in patient fibroblasts and reduced downstream mevalonate and dolichol pathway metabolites in the W6X worms.

Major Comments:
Overall, this manuscript is very well written, the figures and data presentation are clear, and the data appears to support the claims made by the authors. This work is exciting and warrants publication and perhaps eventual clinical investigation in the restorative properties of atorvastatin in this patient population.

Additional recommended studies to further strengthen this manuscript include:

1) Show loss of protein expression in knockout worms. Have the investigators ruled out the presence of an alternative start site expressing an N-terminally truncated protein with partial or dominant negative function? Please comment.

2) Show evidence of genotyping in mutant worms as a supplementary figure.

3) Demonstrate the effect of atorvastatin on healthy fibroblasts to show impact on polyprenol-to-dolichol ratio (or cite literature). This control experiment would help with interpretation of the results from atorvastatin treatment on patient cell lines.

4) Perform serum transferrin isoelectric focusing on wild type fibroblasts and patient cell lines treated with atorvastatin and DMSO vehicle control to show if there is rescue from the typical type I transferrin isoelectric focusing profile of biallelic mutation carriers.

Reviewer #2

(Remarks to the Author)

Reviewer #3

(Remarks to the Author)
This is a well-executed study aimed at identifying a repurposed drug for use in the treatment of SRD5A3-CDG. The screen takes advantage of a newly developed worm model for this disorder, which the authors characterize on multiple levels. The

characterization of the biochemical phenotype and analysis of certain neuronal cell types is viewed as an added strength of the study. The critiques below are intended primarily to improve the strength of their conclusions. The authors are asked to provide new experimental evidence to broaden our view of the possible mechanisms accounting for impaired motility in this model, and better explain the specificity of effects of the hits from the screen. Of primary concern to this reviewer is the conclusion that atrovastatin leads to improved phenotypes through its (indirect) effects on the dolichol/polyprenol ratio, which is not an overly compelling argument based on the evidence provided.

1. The authors have chosen to focus on atorvastatin for the majority of this study, and present evidence that this compound can impact the ratio of polyprenol/dolichol (only in SRD5A3-CDG patient cells). The rationale that a statin (which acts well upstream of the SRD5A3 reaction) would have an impact on polyprenol/dolichol ratios is counterintuitive since one might expect that a reduction in melavonate would limit available lipids for the dolichol pathway. The argument that allowing less substrate through the pathway is beneficial is challenging to make here without the experimental proof that the dolichol/polyprenol ratio is impacted in the worms (an unfortunate limitation that is acknowledged here). Instead, compounds that might shift metabolic flux away from cholesterol biosynthesis and towards dolichol are more likely candidates, based on the flux argument. This was demonstrated by Haeuptle and co-workers (JBC 2011; PMID: 21183681) through their work on zaragozic acid (a squalene synthase inhibitor) in the context of CDG cells. In light of the absence of the cholesterol biosynthesis branch from mevalonate in worms, this same mechanism can't be explored in that model but it would be of interest to ask whether a squalene synthase inhibitor like zaragozic acid could produce the same effects as atorvastatin in the SRD5A3 patient cells, with regard to dolichol/polyprenol ratios.

2. I question whether the positive effect of atorvastatin in the SRD5A3 worm model relates to its ability to instead impact another pathogenic consequence in these animals. The authors should explore whether there is any cholesterol accumulation within the neurons of this model using cholesterol-binding dyes (such as filipin) or by feeding the mutant worms its fluorescent analog, dehydroergosterol (PMC37336) and asking whether neuronal accumulation is observed. It cannot be ruled out that cholesterol buildup is occurring (through an as-yet-defined mechanism) as noted in cells with defects in other parts of the dolichol pathway (e.g. NUS1/DHDDS). Atorvastatin might be acting to reduce this accumulation and improve phenotypes by altering how the exogenous cholesterol is taken up and transported. Addressing this point would strengthen the conclusions around the mechanism of action, especially in the absence of any direct proof that polyprenols are affected.

3. The authors should consider testing whether piroxicam has any effects on the abundance or ratio of dolichol/polyprenol in the patient fibroblasts, considering its beneficial impact in the worm model.
It was somewhat surprising to see positive hits from the screen representing several different classes of drugs. This would seem to suggest that multiple mechanisms of disease may exist. Do these different compounds somehow converge on the dolichol pathway?

4. The authors provide evidence for exacerbated phenotypes in the SRD5A3 worm model in the presence of tunicamycin but do not provide evidence that glycosylation is impaired in this model. Was any attempt made to address whether N-linked glycosylation is impacted? The lack of any effect on glycosylation in this system might highlight a different mechanism of action to account for the abnormal motility observed. I would add another paragraph in the limitations of the study section that states that the full breadth of molecular consequences in this model remain to be characterized and that additional mechanisms affecting the nervous system could explain why multiple different classes of compounds were effective in improving phenotypes.

5. Panel I in Figure 5 is somewhat misleading since no experimental evidence that glycosylation is restored was provided. Along these lines, one highly useful experimental addition to the present work would be to ask whether atorvastatin or piroxicam impacts the sensitivity of the SRD5A3 worm model to tunicamycin. This would again strengthen and support the specificity of effects of these compounds and whether they improve phenotypes via a dolichol/glycosylation-dependent pathway.

Reviewer #4

(Remarks to the Author)
Summary
This manuscript presents a novel C. elegans model of SRD5A3-CDG, using CRISPR to generate srdf-3 loss-of-function alleles. The mutant animals recapitulate key clinical features of the human disorder, including impaired motility, neuronal degeneration, ER stress sensitivity, and metabolic rewiring of the mevalonate pathway. Through a high-throughput drug screen, atorvastatin is identified as a promising repurposing candidate. Cross-species validation using SRD5A3-CDG patient fibroblasts supports the metabolic findings and therapeutic potential of HMGR inhibition. The work is timely, technically sound, and of high translational relevance, but some mechanistic and validation issues require clarification.

Strengths: Precise engineering of W6X and deletion alleles enables rigorous phenotyping in a genetic model system. The work examines a wide array of disease features, from development and neuromuscular function to glycosylation-linked metabolism. Data from patient-derived fibroblasts reinforce the metabolic disruptions observed in worms and confirm clinical relevance. The identification of atorvastatin as a suppressor of srdf-3 phenotypes suggests actionable drug repurposing potential.

Concerns & Recommendations
- Incomplete Validation of Atorvastatin's Mechanism of Action: while the study reports that atorvastatin lowers mevalonate levels and rescues phenotypes, direct evidence that HMG-CoA reductase (HMGR/hmgr-1) is the primary therapeutic target is lacking. No RNAi or mutant analysis of hmgr-1 was performed to mimic or enhance atorvastatin effects. Rescue by polyphenols and NSAIDs, some of which have broad anti-inflammatory or antioxidant effects, raises the possibility that rescue occurs through alternative pathways, such as mitochondrial or immune modulation. I would recommend that genetic validation of HMGR dependence (e.g., hmgr-1 RNAi or mutant), or (at the very least) acknowledge more explicitly in the discussion that HMGR (hmgr-1) is a likely but not definitively proven target of atorvastatin in this context.

- Statistical Reporting: statistical methods, n-values, and exact p-values are inconsistently reported across figures and supplementary data. I would recommend that they ensure consistent annotation of all figures with sample sizes, test types, and significance values.
Overall, this manuscript presents a compelling new model of SRD5A3-CDG, with impressive integration of in vivo, in vitro, and chemical biology approaches. The therapeutic insights into HMGR inhibition are exciting but would benefit from more mechanistic validation. With revisions addressing the points above, particularly target specificity and statistical clarity, this study would make a strong contribution to the fields of glycosylation disorders, metabolic regulation, and therapeutic repurposing.

Recommendation: Accept with Minor Revisions

Reviewer #5

(Remarks to the Author)
Summary

This manuscript reports creation of a C. elegans model for SRD5A3-CDG W91X, characterization of changes in this new model, analysis of patient-derived fibroblasts, and identification of a compound that ameliorates defects (Atorvastatin, an FDA-approved HMG-Co-A reductase inhibitor). The manuscript concludes that Atorvastatin should be considered as a candidate drug to treat SRD5A3-CDG. This manuscript is strong and clearly written. The experiments are well-designed and likely were undertaken rigorously, the results will be of considerable interest to researchers in the field, and the conclusions seem appropriate at most points. However, the manuscript is missing some important details and controls. Occasionally, the manuscript phrasing is confusing or could mislead the reader. Correcting these is important for understanding the results presented, essential for interpretation and conclusions, or needed to conform with the rigorous reporting standards of the journal and the scientific community.

Major comments
The manuscript is confusing when discussing polyprenol-to-dilicol levels. The text frequently states that the ratio decreases, but these changes are not significant when the figures are examined. The manuscript text cannot simply say "decreased" without making this clear, else the reader will misinterpret these results.

Characterization of developmental, physical, neurological, and metabolic defects of the C. elegans W6X loss of function allele is strong, but some important details and comparisons are lacking. The manuscript fails to include the srdf-3(deletion) allele throughout the entirety of the model characterization. Currently, the deletion allele is used only in survival, fecundity, and motility assays, and not in neurological or metabolic defect assays, The latter are arguably more important/relevant to the disease. The manuscript should N2 and srdf-3(deletion) strains in Figure 5 A-D to determine if Atorvastatin has any effects on the control animals.

The manuscript attributes all the changes observed in drug-treated srdf-3 mutant animals to the actions of the drug on C. elegans, not on the co-cultured bacteria they eat. (C. elegans are usually cultured on live E. coli). This underlying assumption needs to be tested by providing experimental results that determine if the drug ameliorates defect when C. elegans are raised on dead E. coli. Related note: The manuscript states "significantly lower glycogen abundance in mutants compared to wild-type worms (Figure 3E), suggesting potential disruptions in glycogen metabolism." The bacteria may be altered metabolically by the drug, leading for differences in their glycogen storage.

Were researchers blinded as to genotype/treatment when capturing/selecting images for analysis or for any scoring that was undertaken manually? An explicit statement in the Methods section is needed.

Description of the screen should be improved for the readers, including clarity about replicates during singlicate screening (in Methods) and providing data for Atorvastatin impact on motility/swimming defects in screen and rescreening steps. Also, more detail about screening methods is needed. What sampling rate for 10 hours? How many samples? This section is very unclear and lacks critical details, including assessment of how well Atorvastatin improves W6X motility.

Minor Comments

Given large difference between C. elegans and humans in how circuits control locomotion, use of the word "mirrors" in the

following sentence implies the same neuromuscular pathways lead to decreased motility across species "This decrease in motility mirrors the progressive movement deficits observed in SRD5A3-CDG patients." The authors might consider rephrasing or clarifying.

"…typically associated with impaired synaptic neurotransmitter release." It is important to note for readers that delayed response to aldicarb can also arise from increased GABA release of function.

Nomenclature for C. elegans and reporting of C. elegans strains is inadequate. A table containing C. elegans strain names and complete genotypes is required. What are the formal allele designations for the srdf-3 alleles? Or were allele designations starting with mod approved formally? For details:
https://wormbase.org/about/userguide/nomenclature#kdge1jhcam89l567bf23i04--10

A precise description of the deletion allele and CRISPR edited allele is required. This can be supplied in supplement, but readers should know precisely what nucleotides were removed or edited in CRISPR editing, including PAM site edits or editing to simplify genotyping

Reference for ufls34[Punc-47p::mCherry] in Methods is missing. Likely this should be referenced as https://doi.org/10.1523/JNEUROSCI.4384-12.2013

For this journal, the "preferred approach is to make data available in repositories"; the source data file is referred to in the text, but this reviewer could not find it for review

CGC needs their preferred acknowledgement statement to be located in the manuscript's Acknowledgement section

It would be helpful if the authors could strengthen the manuscript by demonstrating that srdf-3 disease model defects are ameliorated by decreasing activity of the Atorvastatin target (with a loss-of-function allele or RNAi). They could include a double mutant of W9X and a complete LOF allele of the HMDR ortholog (hmgr-1) or RNAi. This is not essential but would be very useful for confirming identification of the mechanisms of action.

There are minor spelling/grammar errors and citation formatting errors.

In the aldicarb assay, animals are considered paralyzed when they "fail to move" after prodding. This is vague– does pumping/twitching count as movement?

Figure S4 is low quality; this should be higher resolution in the final manuscript

Version 1:

Reviewer comments:

Reviewer #1

(Remarks to the Author)
Accept. No further comments.

Reviewer #2

(Remarks to the Author)

Reviewer #3

(Remarks to the Author)
I thank the authors for their thoughtful responses to all the critiques. It is somewhat discouraging that many of the suggested experiments were unable to be performed but I feel as though the revised manuscript satisfactorily clarifies certain mechanistic concerns that were raised and also more carefully states the primary conclusions of the research. I have no further comments or issues.

Reviewer #4

(Remarks to the Author)

Reviewer #5

(Remarks to the Author)

The author have appropriately addressed all of this reviewer's comments

Open Access This Peer Review File is licensed under a Creative Commons Attribution 4.0 International License, which permits use, sharing, adaptation, distribution and reproduction in any medium or format, as long as you give appropriate credit to the original author(s) and the source, provide a link to the Creative Commons license, and indicate if changes were made.
In cases where reviewers are anonymous, credit should be given to 'Anonymous Referee' and the source.
The images or other third party material in this Peer Review File are included in the article's Creative Commons license, unless indicated otherwise in a credit line to the material. If material is not included in the article's Creative Commons license and your intended use is not permitted by statutory regulation or exceeds the permitted use, you will need to obtain permission directly from the copyright holder.
To view a copy of this license, visit https://creativecommons.org/licenses/by/4.0/

We thank the reviewers for their careful and constructive comments. We have addressed each point in detail and revised the manuscript accordingly.

Reviewer #1

Overall, this manuscript is very well written, the figures and data presentation are clear, and the data appears to support the claims made by the authors. This work is exciting and warrants publication and perhaps eventual clinical investigation in the restorative properties of atorvastatin in this patient population.

1.1. Show loss of protein expression in knockout worms. Have the investigators ruled out the presence of an alternative start site expressing an N-terminally truncated protein with partial or dominant negative function?

Western blotting for endogenous SRDF-3 is not feasible due to the lack of validated antibodies and the difficulty of detecting this low-abundance membrane protein in C. elegans. As an alternative, we quantified srdf-3 mRNA levels. qPCR analysis (Figure S8C) shows complete loss of transcript in the deletion allele and strongly reduced transcript in the W6X allele, consistent with nonsense-mediated decay. Importantly, the W6X and deletion strains show indistinguishable phenotypes, supporting that both are functional nulls and arguing against expression of any truncated protein.

1.2. Show evidence of genotyping in mutant worms as a supplementary figure.

Genotyping validation for both the W6X point mutation and the srdf-3 deletion allele has been added to the Supplementary Information (Figure S8).

1.3. Demonstrate the effect of atorvastatin on healthy fibroblasts to show impact on polyprenol-to-dolichol ratio (or cite literature). This control experiment would help with interpretation of the results from atorvastatin treatment on patient cell lines.

We agree that testing the effect of atorvastatin on healthy control fibroblasts would be informative. Unfortunately, our fibroblast stocks have been exhausted and repeating the LCMS experiment on newly sourced controls is not feasible within the scope of this revision due to limitations in personnel and resources. To our knowledge, there are currently no published studies that quantify polyprenol, dolichol, or their ratios in healthy human fibroblasts following treatment with atorvastatin or other statins.

1.4. Perform serum transferrin isoelectric focusing on wild type fibroblasts and patient cell lines treated with atorvastatin and DMSO vehicle control to show if there is rescue from the typical type I transferrin isoelectric focusing profile of biallelic mutation carriers.

We agree that assessing glycosylation is important for interpreting the functional impact of atorvastatin. However, transferrin is synthesized and glycosylated primarily in the liver and secreted into serum; therefore, assessing transferrin glycosylation in patient fibroblasts is not an appropriate assay for detecting glycosylation changes. Additionally, normal transferrin profiles have been reported in patients with SRD5A3-CDG (Jaman et al., Front Genet, 2021), indicating that serum transferrin is not a sensitive marker of this CDG type in all cases.

Glycoproteomics analysis of lysates from the same SRD5A3-CDG fibroblasts lines used in our study has been published, with results suggesting that cellular glycosylation machinery is altered in these

fibroblasts with SRD5A3-deficiency (Garapati et al., Glycobiology, 2024). We have also added a note in the Discussion of the revised manuscript acknowledging the previously reported glycosylation abnormalities in these fibroblast lines and highlighting that future work evaluating the impact of atorvastatin on glycosylation in SRD5A3-CDG patient lines would be valuable.

Reviewer #2

This is a very interesting project which could help to establish a new therapeutic approach for many CDG patients. The publication is very well written; the experiments are sound and promote the argumentation of the authors. I really enjoyed reading the text and I only have some minor remarks.

2.1. Introduction, page 3, 2nd paragraph: SRD5A3 gene should be in italics.

We thank the reviewer for the correction. SRD5A3 has now been italicized.

2.2. Methods, page 15, 3rd paragraph: E.Coli should be in italics.

This has been corrected.

2.3. Results, page 6, 2nd paragraph: delete 'on day 1'.

This has been corrected.

2.4. Legend to Figure 1B: Please indicate what the three light blue blocks are or could be.

We thank the reviewer for this suggestion. The legend has been updated to indicate that the three light-blue blocks represent predicted transmembrane domains.

Reviewer #3

This is a well-executed study aimed at identifying a repurposed drug for use in the treatment of SRD5A3-CDG. The screen takes advantage of a newly developed worm model for this disorder, which the authors characterize on multiple levels. The characterization of the biochemical phenotype and analysis of certain neuronal cell types is viewed as an added strength of the study.

Weaknesses

1) provide new experimental evidence to broaden our view of the possible mechanisms accounting for impaired motility in this model, and better explain the specificity of effects of the hits from the screen.

2) primary concern is the conclusion that atorvastatin leads to improved phenotypes through its (indirect) effects on the dolichol/polyprenol ratio, which is not an overly compelling argument based on the evidence provided.

Comments

3.1. Rational that a statin would have an impact on polyprenol/dolichol ratios is counterintuitive since one might expect that a reduction in mevalonate would limit available lipids for the dolichol pathway.

- The argument that allowing less substrate through the pathway is beneficial is challenging to make here without the experimental proof that the dolichol/polyprenol ratio is impacted in the worms (an unfortunate limitation that is acknowledged here).
- Compounds that might shift metabolic flux away from cholesterol biosynthesis and towards dolichol are more likely candidates, based on the flux argument.
- This was demonstrated by Haeuptle and co-workers (JBC 2011; PMID: 21183681) through their work on zaragozic acid (a squalene synthase inhibitor) in the context of CDG cells.

- Zaragozic acid mechanism can't be explored in the worm, but it would be of interest to ask whether a squalene synthase inhibitor like zaragozic acid could produce the same effects as atorvastatin in the SRD5A3 patient cells, with regard to dolichol/polyprenol ratios."

We thank the reviewer for this insightful comment. In the revised manuscript, we now include new data generated using a dedicated targeted LC–MS/MS assay optimized for polyprenol and dolichol quantification in W6X worms (Figures 3A–C, 5C, and S9). These data confirm that W6X mutants exhibit elevated polyprenols, reduced dolichols, and an increased polyprenol:dolichol ratio compared to wild type; however, atorvastatin treatment did not significantly alter these metabolites in worms. We have clarified this in the Results and expanded the Discussion to explain that phenotypic rescue in worms occurs independently of polyprenol/dolichol normalization. We also discuss the flux-redirecting strategy reported by Haeuptle et al. in the context of SRD5A3 and dolichol disorders, and we incorporate the ratio-based disease mechanism proposed by Wilson et al. (2024) to highlight why different points of mevalonate-pathway modulation may have divergent effects on dolichol disorders.

3.2. Does the positive effect of atorvastatin in the SRD5A3 worm model relate to its ability to instead impact another pathogenic consequence in these animals?

- Explore whether there is any cholesterol accumulation within the neurons of this model using cholesterol-binding dyes (such as filipin) or by feeding the mutant worms its fluorescent analog, dehydroergosterol (PMC37336) and asking whether neuronal accumulation is observed. It cannot be ruled out that cholesterol buildup is occurring (through an as-yet-defined mechanism) as noted in cells with defects in other parts of the dolichol pathway (e.g. NUS1/DHDDS).
- Atorvastatin might be acting to reduce this accumulation and improve phenotypes by altering how the exogenous cholesterol is taken up and transported. Addressing this point would strengthen the conclusions around the mechanism of action, especially in the absence of any direct proof that polyprenols are affected."

We appreciate the reviewer's suggestion. However, C. elegans does not synthesize cholesterol and relies entirely on exogenous dietary cholesterol. Because worms lack endogenous sterol biosynthesis and do not accumulate cholesterol in a mammalian-like manner, assays such as filipin staining or dehydroergosterol feeding would not provide meaningful mechanistic insight in this model. For this reason, cholesterol accumulation is unlikely to contribute to the phenotypes or to the rescue observed with atorvastatin.

3.3. Test whether piroxicam has any effects on the abundance or ratio of dolichol/polyprenol in the patient fibroblasts.

We agree that examining the effects of piroxicam on polyprenol and dolichol levels in patient fibroblasts would be interesting. However, our study is focused on validating atorvastatin as a candidate therapeutic for SRD5A3-CDG, and extending LC–MS/MS analyses to additional compounds is beyond the scope of the current work due to resource and budget constraints.

3.4. Surprising to see positive hits from the screen representing several different classes of drugs. This would seem to suggest that multiple mechanisms of disease may exist. Do these different compounds somehow converge on the dolichol pathway?

We thank the reviewer for this observation. The compounds identified in our screen span several pharmacological classes, which likely reflects that SRD5A3 deficiency perturbs multiple downstream cellular processes, rather than a single pathway. These compounds do not necessarily converge on the

dolichol pathway; instead, they may act through distinct mechanisms that improve the stress-sensitive phenotypes observed in the worm model. We have clarified this point in the Discussion.

3.5. Was any attempt made to address whether N-linked glycosylation is impacted? The lack of any effect on glycosylation in this system might highlight a different mechanism of action to account for the abnormal motility observed. I would add another paragraph in the limitations of the study section that states that the full breadth of molecular consequences in this model remain to be characterized and that additional mechanisms affecting the nervous system could explain why multiple different classes of compounds were effective in improving phenotypes.

We agree that assessing N-linked glycosylation is important for understanding the consequences of SRD5A3 deficiency. In our study, we evaluated tunicamycin sensitivity as an indirect functional readout, and W6X mutants showed increased sensitivity, consistent with impaired N-glycosylation capacity. However, we acknowledge that this assay does not directly measure glycosylation status. We have now added a statement to the Limitations section noting that the full range of molecular consequences in the worm model remains to be characterized, and that additional mechanisms may contribute to the observed phenotypes.

3.6. Panel I in Figure 5 is somewhat misleading since no experimental evidence that glycosylation is restored was provided

The legend for Figure 5I has been revised to clearly indicate that the schematic represents a hypothetical model and not experimental evidence of restored glycosylation.

3.7. Re: glycosylation restoration hypothesis: One highly useful experimental addition to the present work would be to ask whether atorvastatin or piroxicam impacts the sensitivity of the SRD5A3 worm model to tunicamycin. This would again strengthen and support the specificity of effects of these compounds and whether they improve phenotypes via a dolichol/glycosylation-dependent pathway.

We appreciate the reviewer's suggestion. However, W6X mutants are already hypersensitive to tunicamycin at the concentrations required to reveal an N-glycosylation defect, resulting in severe developmental arrest. Under these conditions, co-treatment with atorvastatin or piroxicam would not yield interpretable results, as tunicamycin-induced toxicity overwhelms the phenotype.

Reviewer #4
1) The work is timely, technically sound, and of high translational relevance
2) Precise engineering of W6X and deletion alleles enables rigorous phenotyping in a genetic model system
3) the work examines a wide array of disease features, from development and neuromuscular function to glycosylation-linked metabolism
4) Data from patient-derived fibroblasts reinforce the metabolic disruptions observed in worms and confirm clinical relevance.
5) The identification of atorvastatin as a suppressor of srdf-3 phenotypes suggests actionable drug repurposing potential.

Overall, this manuscript presents a compelling new model of SRD5A3-CDG, with impressive integration of in vivo, in vitro, and chemical biology approaches. The therapeutic insights into HMGR inhibition are exciting but would benefit from more mechanistic validation. With revisions addressing the points above, particularly target specificity and statistical clarity, this study would make a strong contribution to the fields of glycosylation disorders, metabolic regulation, and therapeutic repurposing.

Weaknesses
Some mechanistic and validation issues require clarification.
1) The therapeutic insights into HMGR inhibition would benefit from more mechanistic validation
2) With revisions addressing the points above, particularly target specificity and statistical clarity

Comments
4.1. Direct evidence that HMG-CoA reductase (HMGR/hmgr-1) is the primary therapeutic target is lacking. No RNAi or mutant analysis of hmgr-1 was performed to mimic or enhance atorvastatin effects.
- Recommend that genetic validation of HMGR dependence (e.g., hmgr-1 RNAi or mutant), or (at the very least) acknowledge more explicitly in the discussion that HMGR (hmgr-1) is a likely but not definitively proven target of atorvastatin in this context.

We agree that genetic validation of HMGR dependence would strengthen the mechanistic interpretation. We attempted RNAi knockdown of hmgr-1 (F08F8.2), but as reported by Ranji et al. (PLoS One, 2014), hmgr-1 loss is lethal in C. elegans*, and we similarly observed embryonic/larval lethality that prevented phenotypic assessment. The hmgr-1 deletion allele from the CGC (strain DG4384) is also annotated as lethal. However, Ranji et al. showed that hmgr-1 loss phenocopies statin treatment and that both effects are rescued by mevalonate, establishing hmgr-1 as the statin target in* C. elegans*. We have now noted in the Discussion that HMGR is the most likely, although not genetically confirmed, target of atorvastatin in this model due to lethality of* hmgr-1 *loss.*

4.2. Statistical methods, n-values, and p-values are inconsistently reported across figures and supplementary data. Ensure consistent annotation of all figures with sample sizes, test types, and significance values.

We thank the reviewer for bringing this to our attention. All figures and supplementary figures have now been updated to include consistent reporting.

Reviewer #5

1) This manuscript is strong and clearly written.
2) The experiments are well-designed and likely were undertaken rigorously, the results will be of considerable interest to researchers in the field, and the conclusions seem appropriate at most points.

Weaknesses
1) missing some important details and controls.
2) Occasionally, the manuscript phrasing is confusing or could mislead the reader. Correcting these is important for understanding the results presented, essential for interpretation and conclusions, or needed to conform with the rigorous reporting standards of the journal and the scientific community

Comments
5.1. The manuscript is confusing when discussing polyprenol-to-dolichol levels. The text frequently states that the ratio decreases, but these changes are not significant when the figures are examined. The manuscript text cannot simply say "decreased" without making this clear, else the reader will misinterpret these results

We agree with the reviewer that using terms such as 'increased' or 'decreased' can misinterpret the results as significance threshold were not meet ($p<0.05$), despite of increased fold-change. In the revised manuscript, we modified the language to 'directionally higher or lower' with fold-change (effect size; e.g. 4-fold) and p-value.

5.2. The manuscript fails to include the *srdf-3(deletion)* allele throughout the entirety of the model characterization. Currently, the deletion allele is used only in survival, fecundity, and motility assays, and not in neurological or metabolic defect assays, The latter are arguably more important/relevant to the disease.

The srdf-3 deletion allele has now been added to the motility and neuronal assays, and these data are included as Figure S6.

5.3. Manuscript should include N2 and *srdf-3(deletion)* strains in Figure 5 A-D to determine if Atorvastatin has any effects on the control animals."

We appreciate the reviewer's suggestion. Because our study focuses on the patient-specific W6X allele, and due to resource constraints, we prioritized metabolomic and rescue analyses in this mutant. However, untreated N2 data are provided in Figure 3 as the control reference, and both N2 and the deletion allele are fully characterized in earlier phenotypic assays.

5.4. The manuscript attributes all the changes observed in drug-treated srdf-3 mutant animals to the actions of the drug on C. elegans, not on the co-cultured bacteria they eat. (C. elegans are usually cultured on live E. coli). This underlying assumption needs to be tested by providing experimental results that determine if the drug ameliorates defect when C. elegans are raised on dead E. coli.

We thank the reviewer for raising this control. We have now tested whether the rescue depends on effects on the co-cultured bacteria. As shown in Figure S7, atorvastatin rescues the progeny defect in W6X mutants even when animals are raised on heat-killed E. coli, indicating that the rescue is independent of bacterial viability and reflects a direct effect on the worms.

5.5. The manuscript states “significantly lower glycogen abundance in mutants compared to wild-type worms (Figure 3E), suggesting potential disruptions in glycogen metabolism.” The bacteria may be altered metabolically by the drug, leading for differences in their glycogen storage.

We thank the reviewer for this comment. The glycogen measurements in Figure 3E were performed as part of the baseline characterization of the W6X mutant and were not conducted under drug-treated conditions, so this phenotype is not attributed to atorvastatin or to bacterial metabolism. In addition, in Figure S7, we now show that atorvastatin rescues the progeny defect even when animals are raised on heat-killed E. coli*, indicating that the drug's effects are not dependent on changes in bacterial metabolism.*

5.6. Were researchers blinded as to genotype/treatment during imaging or scoring? Explicit statement needed.

Researchers were blinded to genotype and treatment during imaging and scoring. We have added an explicit statement describing the blinding procedures in the Methods section of the revised manuscript.

5.7. Clarify aldicarb scoring: does failure to move mean no pumping/twitching or just failure to crawl? Description of the screen should be improved for the readers, including clarity about replicates during singlicate screening (in Methods) and providing data for Atorvastatin impact on motility/swimming defects in screen and rescreening steps. Also, more detail about screening methods is needed. What sampling rate for 10 hours? How many samples? This section is very unclear and lacks critical details, including assessment of how well Atorvastatin improves W6X motility.

We thank the reviewer for these helpful suggestions. In the aldicarb assay, “failure to move” refers specifically to failure to crawl. We have clarified this definition in the Methods. We also expanded the Methods section to provide additional detail on the screening workflow. Finally, we added a brief description of atorvastatin's rescue effect in both the primary and secondary screens in the Results section (Z-scores).

5.8. It would be helpful if the authors could strengthen the manuscript by demonstrating that srdf-3 disease model defects are ameliorated by decreasing activity of the Atorvastatin target (with a loss-of-function allele or RNAi). They could include a double mutant of W9X and a complete LOF allele of the HMDR ortholog (hmgr-1) or RNAi. This is not essential but would be very useful for confirming identification of the mechanisms of action.

We agree that reducing hmgr-1 activity genetically would be a valuable way to further validate the mechanism of action. However, hmgr-1 knockdown by RNAi resulted in embryonic/larval lethality in our hands, and the hmgr-1 deletion allele available from the CGC (strain DG4384) is also annotated as lethal. These findings are consistent with published work showing that loss of HMG-CoA reductase function (hmgr-1 mutant) is inviable in C. elegans (Ranji et al., PLoS One, 2014). Therefore, generating double mutants or performing rescue assays under hmgr-1 loss-of-function conditions is not experimentally feasible.

5.9. Given the large difference between C. elegans and humans in how circuits control locomotion, use of the word "mirrors" in the following sentence implies the same neuromuscular pathways lead to

decreased motility across species "This decrease in motility mirrors the progressive movement deficits observed in SRD5A3-CDG patients." The authors might consider rephrasing or clarifying.

We have revised the sentence to replace "mirrors" with "is analogous to," which avoids implying conserved neuromuscular circuitry between humans and C. elegans.

5.10. "…typically associated with impaired synaptic neurotransmitter release." It is important to note for readers that delayed response to aldicarb can also arise from increased GABA release of function.

We have revised the text to clarify that aldicarb resistance can result from either increased GABA release or reduced acetylcholine release.

5.11. A table containing *C. elegans* strain names and complete genotypes is required. What are the formal allele designations for the srdf-3 alleles? Or were allele designations starting with mod approved formally? For details: https://wormbase.org/about/userguide/nomenclature#kdge1jhcam891567bf23i04--10

We thank the reviewer for this feedback. We have now added a table listing all C. elegans strains used in the study, including full genotypes and formal allele designations, as Table S3 in the Supplementary Information.

5.12. A precise description of the deletion allele and CRISPR edited allele is required. This can be supplied in supplement, but readers should know precisely what nucleotides were removed or edited in CRISPR editing, including PAM site edits or editing to simplify genotyping

The details of the CRISPR edited alleles have now been included in Figure S8.

5.13. missing reference for ufIs34[Punc-47p::mCherry] strain in the methods

We have now added a reference for the strain in the methods (Labarre, A, et al. 2021).

5.14. CGC needs their preferred acknowledgement statement to be located in the manuscript's Acknowledgement section

We thank the reviewer for noting this. CGC acknowledgment has now been added to the Acknowledgments section of the revised manuscript.

5.15. Fix typos, citation formatting issues, improve resolution of Figure S4.

We have fixed the typos, formatting and confirmed that Figure S4 is high-resolution.

5.16. Add source data files to final submission

Source data will be provided upon acceptance and made available with the final published version of the manuscript.
