# Supplementary material for: Repurposing the HMG-CoA reductase inhibitor atorvastatin for SRD5A3-congenital disorder of glycosylation
Source: Commun Med (Lond). 2026 Jul 29;6:517. doi: 10.1038/s43856-026-01791-4 (PMC13642943; doi:10.1038/s43856-026-01791-4)
Supplement: Supplementary file 2 [file 43856_2026_1791_MOESM2_ESM.pdf]

Supplementary Text

Liquid chromatography-tandem mass spectrometry (LC-MS/MS) for metabolites in worms

Targeted LC–MS/MS profiling of mevalonate-pathway metabolites and polyisoprenoids was performed at The Metabolomics Innovation Center (TMIC). TMIC used targeted LC-MS/MS workflows optimized for quantification of HMG-CoA, mevalonate, acyl-CoA intermediates, isoprenyl phosphates, and ubiquinones, each employing stable-isotope internal standards and external calibration curves. A dedicated targeted assay was used for quantitation of long-chain polyprenols and dolichols. All metabolite concentrations were normalized to total protein. Metabolite measurements were acquired in two independent runs; within each run data were normalized to the corresponding wild-type controls and then pooled from downstream analysis.

1. Quantitation of HMG-CoA and isoprenyl phosphates

Clear supernatant was mixed 1:1:1 (v/v/v) with HMG-CoA-D3 internal standard solution and dichloromethane (DCM), vortexed for 30 s and centrifuged for 5 min. The upper aqueous phase was dried under nitrogen, then reconstituted in 50 µL of 50% methanol. Calibration standards containing HMG-CoA, acetoacetyl-CoA, acetyl-CoA, and isoprenyl phosphates were prepared by serial dilution in a ratio of 1 to 5 (v/v). Samples and calibration standards (15 µL) were analyzed by MRM-based targeted metabolomics using an Acquity UPLC (*Waters*) coupled to a QTRAP 6500 mass spectrometer (*Sciex)* operated in negative-ion mode, with a C_{18} column (2.1×100 mm, 1.7 µm) and a mobile phase of 2mM ammonium acetate (A) and acetonitrile (B) under a binary gradient (2% to 60% B in 12 min) at 0.35 mL/min and 50 °C. Concentrations of the detected compounds were calculated by interpolating the constructed linear-regression, internal-calibration curves with the peak area ratios measured from sample solutions. IPP and DMAPP co-eluted; total peak area is reported.

2. Quantitation of ubiquinones

Supernatants were extracted twice using a mixture of 250 µL 50% methanol and 500 µL dichloromethane (DCM) (1:1:2, v/v/v). The pooled DCM layers were dried under nitrogen and reconstituted in 50 µL isopropanol. Calibration standards for MK-4, CoQ8, CoQ9, and CoQ10 mixture of dolichols 13-21 mixture of polyprenols 13-21, were prepared in isopropanol parallel by serial dilution in a ratio of 1 to 5 (v/v) as part of TMIC's standard calibration mixture. Only ubiquinones were analyzed for this study: although MK-4 and long-chain polyprenol/dolichol standards were included in the TMIC calibration mixture, MK-4 is not produced by C. elegans and polyprenols were below the detection limit in this panel. Samples and calibration standards (20 µL) were analyzed by LC-MRM/MS using an Acquity UPLC (*Waters*) coupled to a QTRAP 6500 mass spectrometer (*Sciex*) in positive-ion mode, with a C_8 column (2.1× 50 mm, 1.7 µm) and a mobile phase of 0.1% formic acid in water (A) and 0.1% formic acid in isopropanol (B) under a binary gradient (50% to 95% B in 15 min) at 0.25 mL/min and 50 °C. Concentrations of the detected compounds were calculated by interpolating the constructed linear-regression calibration curves from the injection of calibration solutions, with the peak areas measured from sample solutions.

3.. Quantitation of polyprenols and dolichols

Calibration standards were prepared by serial dilution of dolichol (C13–C21) and polyprenol (C13–C21) standards in methanol–chloroform (1:1, v/v). Frozen worm pellets were thawed, diluted (500 µL water per 50 mg tissue), and homogenized at 30 Hz for 3 min using an MM400 mixer mill**.** Two sequential hexane extractions (400 µL each) were performed; pooled organic phases were dried under nitrogen and reconstituted in 30 µL methanol–chloroform (1:1)**.** Protein in the residual aqueous pellet was measured by BCA assay for normalization. Samples and

calibration standards (12 µL) were analyzed by LC–MRM/MS using an Agilent 1290 UHPLC coupled to a Triple Quadrupole 7500 mass spectrometer (*Sciex*), in positive-ion mode, a C_8 UPLC column (2.1× 50 mm, 1.8 µm) and a mobile phase of 2mM ammonium acetate with 0.04% acetic acid in water (A) and a 2mM ammonium acetate with 0.04% acetic acid in acetonitrile:isopropanol:water (300:700:2) (B) under a binary gradient (50% to 75% B from 0-10 min; 75% to 100% B from 10-22 min; 100% B for 1 min) at) at 0.3 mL/min and 55 °C. The column was reconditioned at 50% B for 4.5min between injections. Peak areas were integrated. The concentrations of total dolichols and total polyprenols detected in the samples were calculated with external standard calibration.

LC-MS/MS for analysis of dolichols and polyprenols from fibroblasts

Dolichols and polyprenols were analyzed from unaffected control and patient fibroblasts using Vanquish Horizon UHPLC (*Thermo Fisher Scientific*) coupled to an Orbitrap Fusion Tribrid ID-X mass spectrometer (*Thermo Fisher Scientific*). A 10 min-long targeted method was developed using dolichol and polyprenol standards (*Avanti Polar Lipids*). Extracted lipids were loaded onto a Hypersil Gold Vanquish C_{18} UHPLC column (2.1 × 150mm, 1.9 µm) with flow rate of 300 µl/min. Under a binary gradient of mobile phase A (H_2O:acetonitrile=4:6 (v/v) with 1 mM ammonium acetate) and mobile phase B (isopropanol:methanol:acetonitrile=8:1:1 (v/v/v) with 1 mM ammonium acetate), separation of dolichols and polyprenols was carried out. Mobile phase B from increased from 60 to 85% over 1 min, 85 to 100% over 4 min and maintained at 100% for 2 min. Then, the mobile phase B was decreased from 100 to 40% over 0.1 min and equilibrated at 40% for 2.9 min for the next injection. The column temperature was set at 50°C throughout the analysis. Precursor scans were acquired in the range of 500 to 1,800 m/z at 60,000 resolution (400

m/z) and targeted MS/MS scans at collision energy of 25% were acquired from the precursor m/z of dolichols and polyprenols at 15,000 resolution (400 m/z). Automatic gain control settings for MS and MS/MS were set at 4×10^5 and 5×10^4, respectively, with injection times to reach automatic gain control were set at 50 ms and 22 ms, respectively. For the analysis of dolichols and polyprenols from patient fibroblasts, both untreated and treated with atorvastatin, LC-MS/MS data acquisition was performed using an Orbitrap Fusion Tribrid IQ-X mass spectrometer (*Thermo Fisher Scientific*).

Supplementary Figures and Tables

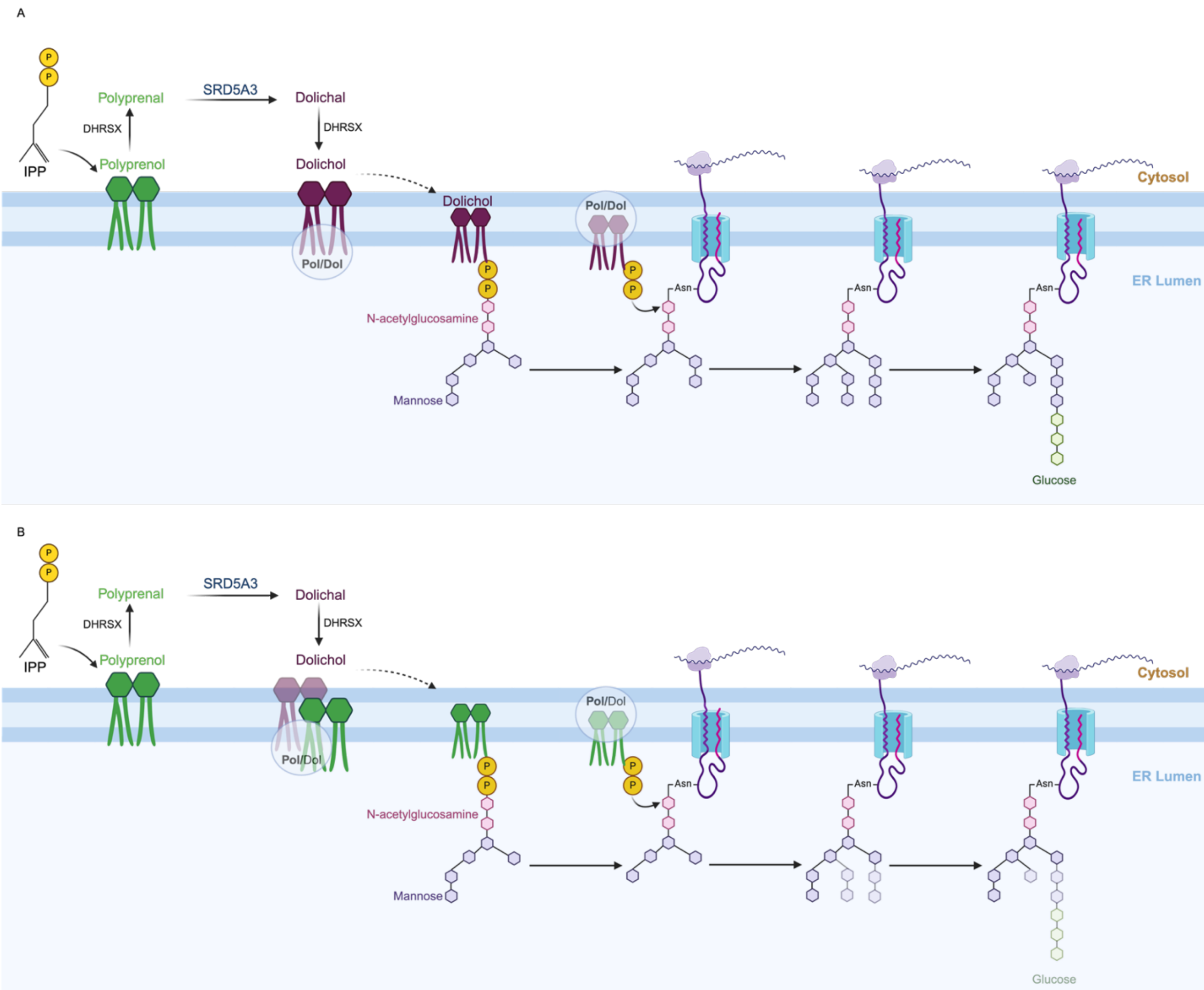

Figure S1. Role of SRD5A3 in glycosylation and disease pathogenesis. (**A**) N-glycosylation is a post-translational modification where glycans are attached to asparagine residues on proteins in the endoplasmic reticulum. Dolichol-phosphate is a lipid carrier of the oligosaccharide prior to its transfer to proteins. Dolichol is synthesized through a multi-step process: DHRSX functions as both a dehydrogenase and reductase, while SRD5A3 catalyzes the reduction of polyprenal to dolichal. (**B**) SRD5A3-CDG is caused by mutations in the SRD5A3 gene, leading to the accumulation of polyprenal and polyprenol. Phosphorylated polyprenol competes with dolichol as

an oligosaccharide acceptor, resulting in inefficient glycosylation and the transfer of immature glycans to proteins. *Created with BioRender.com*

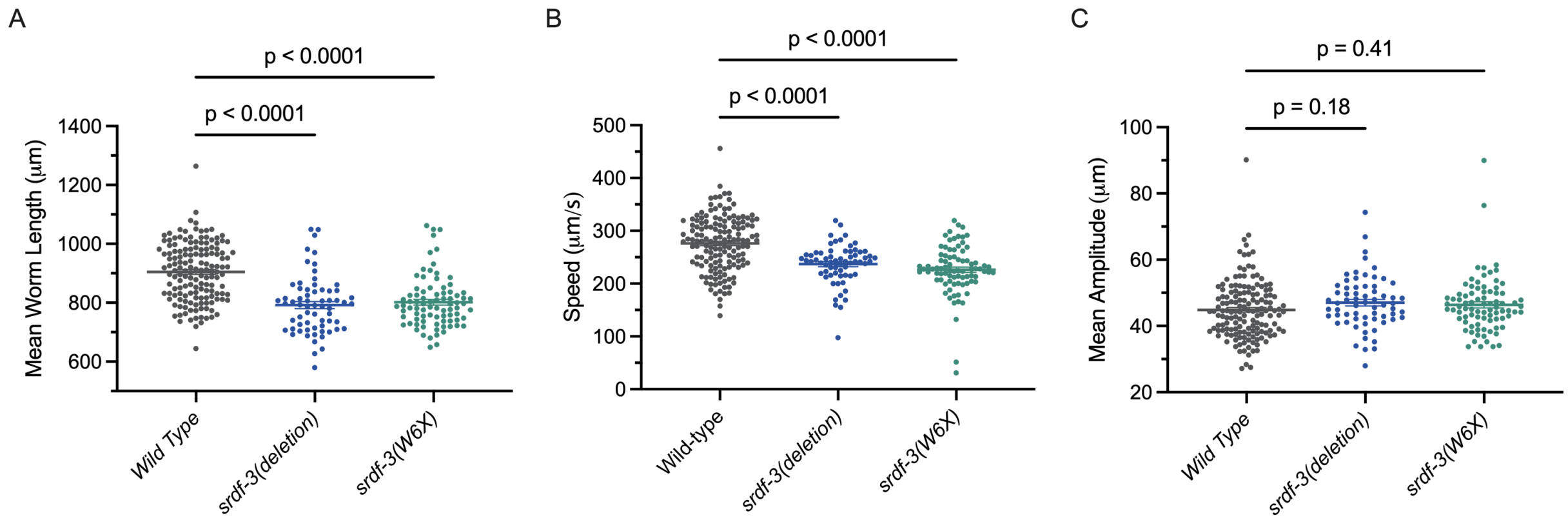

Figure S2. Motility phenotypes of *srdf-3* mutant worms. *srdf-3* deletion and W6X mutant worms exhibit reduced length (**A**) and speed (**B**) but not amplitude (**C**) relative to wild-type worms. *n* = 151 wild-type, *n* = 63 *srdf-3*(deletion), and *n* = 83 *srdf-3*(W6X) worms, pooled from 3 independent experiments. One-way ANOVA with Bonferroni *post hoc* test. Data are presented as mean ± SEM. *p* values are indicated in the figure.

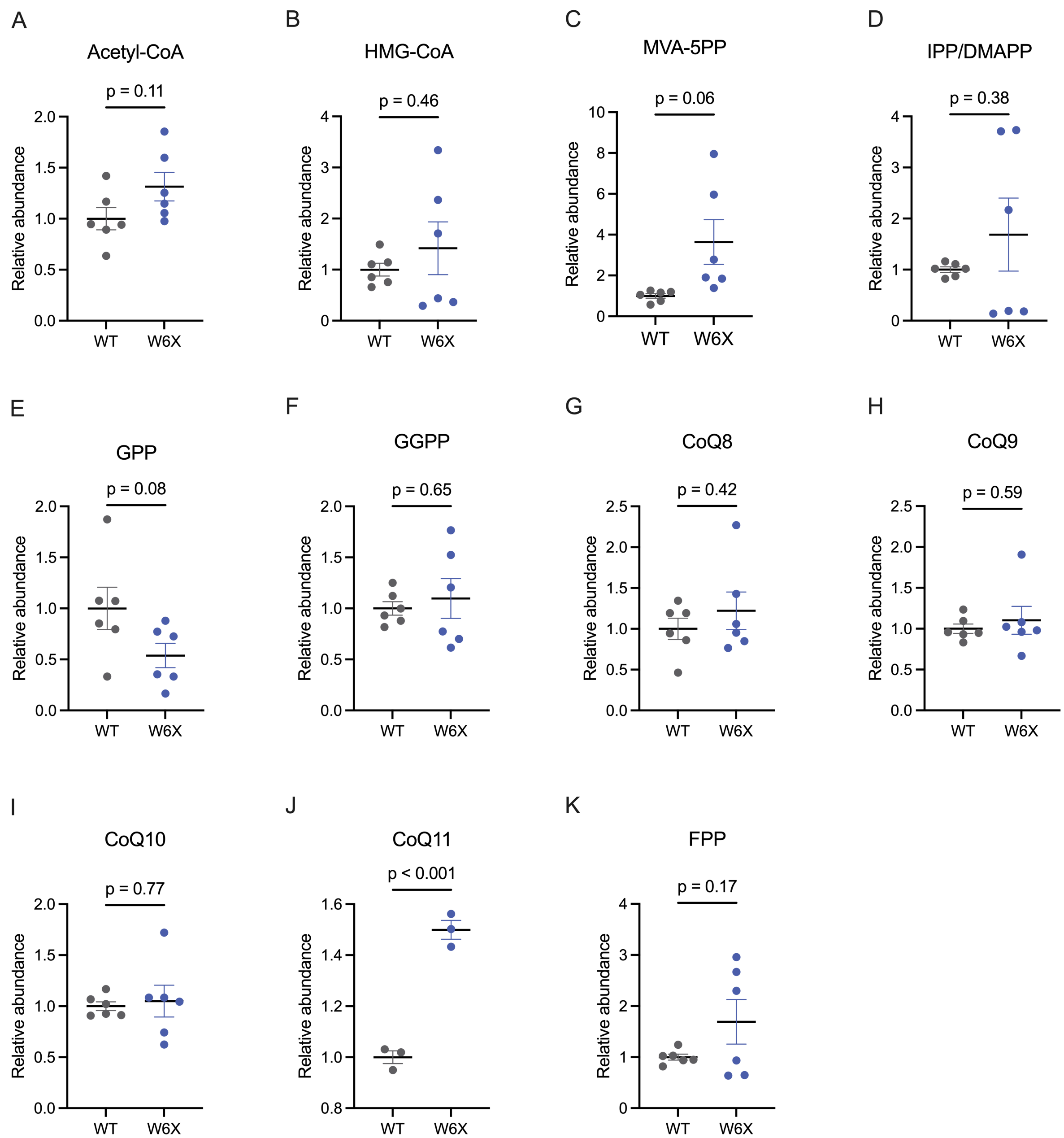

Figure S3. Metabolomic profiling of mevalonate and dolichol biosynthesis pathway metabolites in *srdf-3* W6X mutant worms. (**A-K**) LC-MS/MS quantified metabolites in W6X worms compared to wild-type (WT) animals. For CoQ11, $n = 3$ independent samples per genotype. For all other metabolites, $n = 6$ independent samples per genotype. Two-sided unpaired t-test with

Welch's Correction applied where appropriate. Data are presented as mean ± SEM. p values are indicated in the figure.

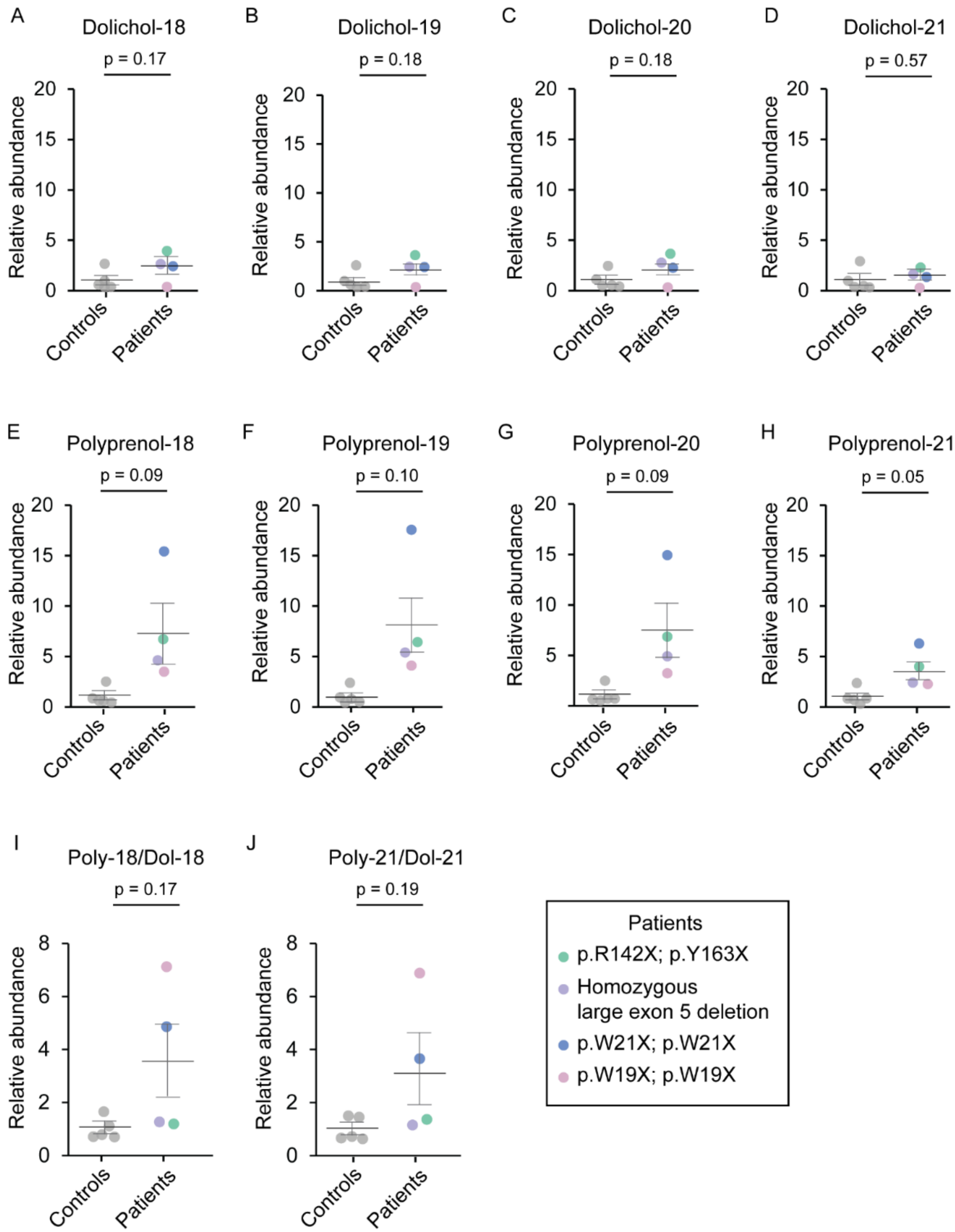

Figure S4. Analysis of dolichols and polyprenols levels in SRD5A3-CDG patient and control fibroblasts. Relative abundance of (**A**) dolichol-18, (**B**) dolichol-19, (**C**) dolichol-20, (**D**) dolichol-

21, (**E**) polyprenol-18, (**F**) polyprenol-19, (**G**) polyprenol-20, and (**H**) polyprenol-21 to unaffected controls, quantified by LC-MS/MS. Ratios of (**I**) polyprenol-18 to dolichol-18 and (**J**) polyprenol-21 to dolichol-21 are shown. Patient genotypes are indicated. For all panels, n = 5 control and n = 4 SRD5A3-CDG independent fibroblast lines. Two-sided unpaired t-test with Welch's Correction applied where appropriate. Data are presented as mean ± SEM. p values are indicated in the figure.

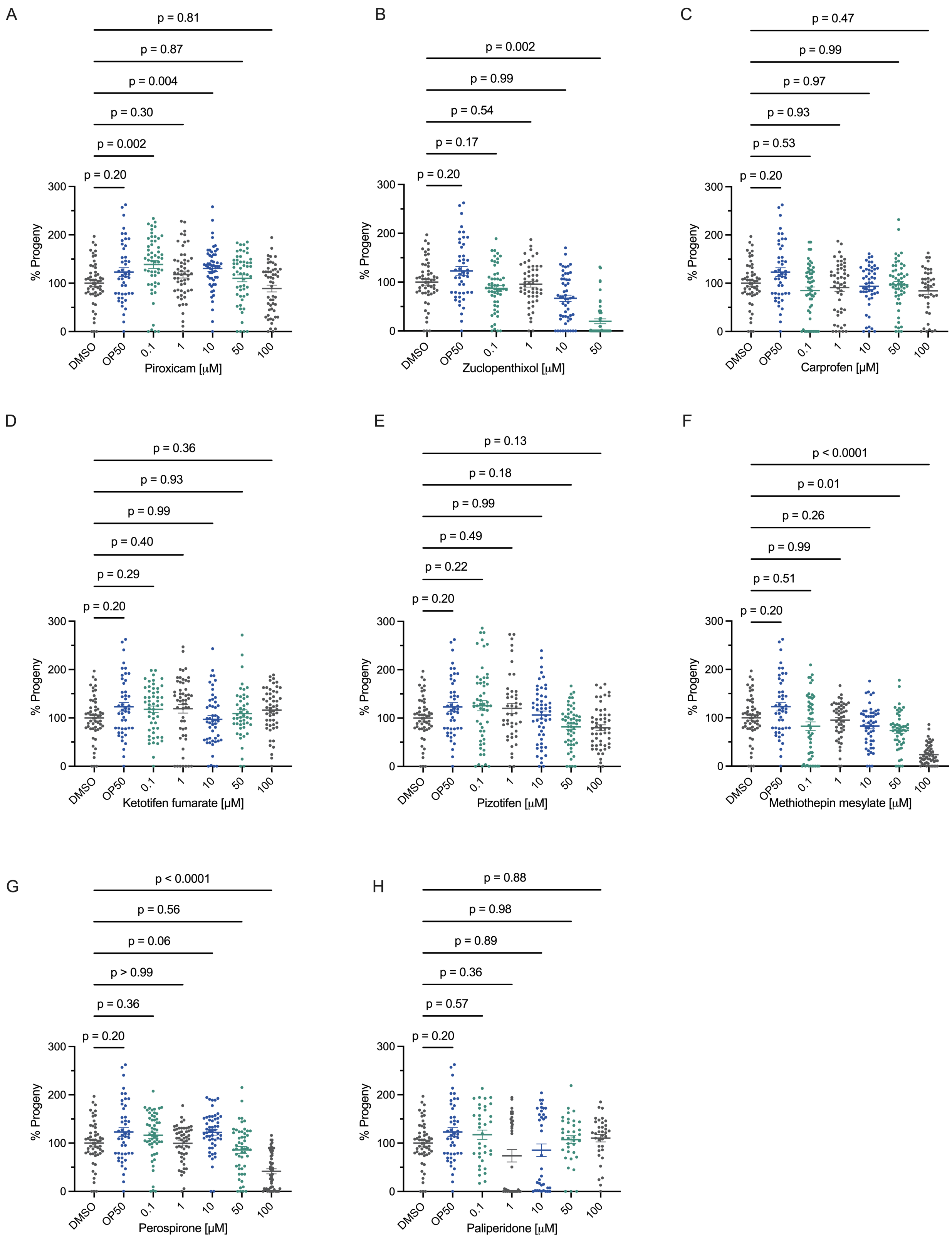

A
p = 0.81
p = 0.87
p = 0.004
p = 0.30
p = 0.002
p = 0.20
% Progeny
DMSO
OP50
0.1
1
10
50
100
Piroxicam [µM]
B
p = 0.002
p = 0.99
p = 0.54
p = 0.17
p = 0.20
% Progeny
DMSO
OP50
0.1
1
10
50
Zuclopenthixol [µM]
C
p = 0.47
p = 0.99
p = 0.97
p = 0.93
p = 0.53
p = 0.20
% Progeny
DMSO
OP50
0.1
1
10
50
100
Carprofen [µM]
D
p = 0.36
p = 0.93
p = 0.99
p = 0.40
p = 0.29
p = 0.20
% Progeny
DMSO
OP50
0.1
1
10
50
100
Ketotifen fumarate [µM]
E
p = 0.13
p = 0.18
p = 0.99
p = 0.49
p = 0.22
p = 0.20
% Progeny
DMSO
OP50
0.1
1
10
50
100
Pizotifen [µM]
F
p < 0.0001
p = 0.01
p = 0.26
p = 0.99
p = 0.51
p = 0.20
% Progeny
DMSO
OP50
0.1
1
10
50
100
Methiothepin mesylate [µM]
G
p < 0.0001
p = 0.56
p = 0.06
p > 0.99
p = 0.36
p = 0.20
% Progeny
DMSO
OP50
0.1
1
10
50
100
Perospirone [µM]
H
p = 0.88
p = 0.98
p = 0.89
p = 0.36
p = 0.57
p = 0.20
% Progeny
DMSO
OP50
0.1
1
10
50
100
Paliperidone [µM]

Figure S5. Dose-response effects of compounds on progeny numbers in *srdf-3* W6X mutant worms. (A) Piroxicam treatment rescues the progeny phenotype of W6X worms at 1 μM and 10 μM doses. One-way ANOVA with Bonferroni *post hoc* test. Data are presented as mean ± SEM. Values are normalized to the mean of the vehicle control. **(B-H)** Dose-response effect of selected compounds on the progeny phenotype. One-way ANOVA with Bonferroni *post hoc* test. Data are presented as mean ± SEM For panels **A–H**, *n* represents biologically independent worms (18 worms per condition per experiment across three independent experiments; exact *n* values for each condition are provided in the source data file **Supplementary Data 6**). Vehicle-control "DMSO"; Bacterial food source control "OP50". *p* values are indicated in the figure.

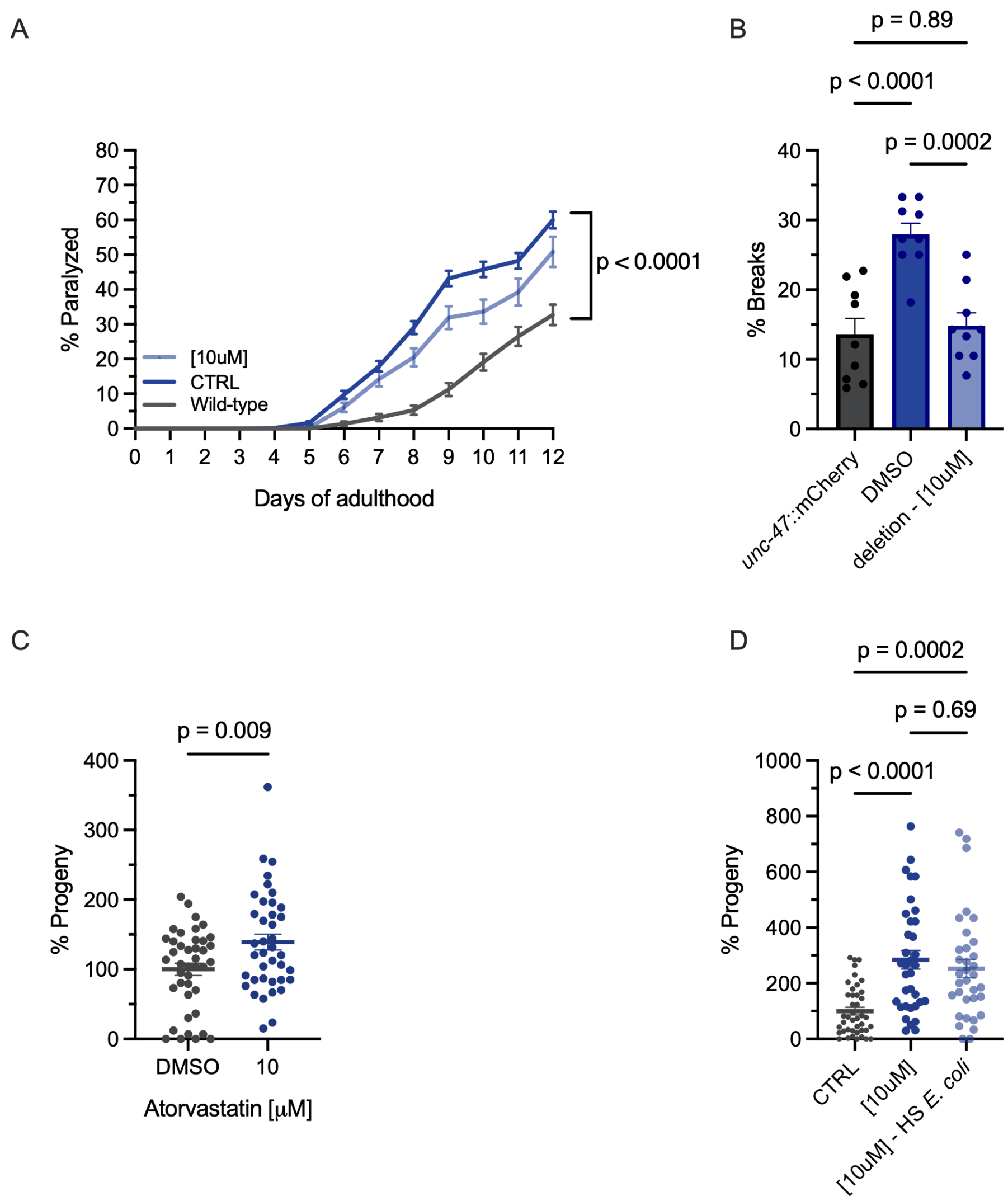

Figure S6. Impaired movement and neuronal function in *srdf-3* deletion worms. (A) *srdf-3* deletion mutants cultured on solid media show increased paralysis compared to wild-type controls. Atorvastatin (10 µM) rescues age-dependent paralysis in *srdf*-3(deletion) worms. *n* = 360 worms per group, pooled across 3 independent experiments. Log-rank (Mantel-Cox) test, two-sided. Data are presented as the mean proportion of paralyzed animals at each timepoint ± SEM. Underlying data from individual experimental replicates are provided in **Supplementary Data 7**. **(B)** Deletion mutants have a higher frequency of breaks/gaps along neuronal processes compared to wild-type *unc-47::*mCherry controls at day 3 of adulthood which is rescued with atorvastatin treatment (10

μM). *n* = 9 plates per group. Brown-Forsythe one-way ANOVA with Dunnett *post hoc* test, two sided. Data are presented as mean ± SEM. **(C)** Atorvastatin (10 μM) rescues the progeny phenotype in *srdf*-3(deletion) mutants. *n* = 41 worms per group, pooled across 3 independent experiments. Values are normalized to the mean of the deletion mutant DMSO vehicle control. Two-sided unpaired t-test. Data are presented as mean ± SEM. **(D)** Heat-shock killed *E. coli* culture does not impact the rescue effect of atorvastatin (10 μM) treatment on the progeny phenotype in *srdf*-3(deletion) mutants *n* = 42 (CTRL; deletion mutant + DMSO), 36 (deletion mutant + 10 μM atorvastatin), and 35 (10 μM–HS *E. coli*; deletion mutant + atorvastatin with heat-shock-killed bacteria) worms pooled across 3 independent experiments. Brown-Forsythe one-way ANOVA with Tukey *post hoc* test. Data are presented as mean ± SEM. Values are normalized to the mean of the OP50 control. *p* values are indicated in the figure. Vehicle-control "DMSO"; Bacterial food source control "OP50". Heat-shock "HS".

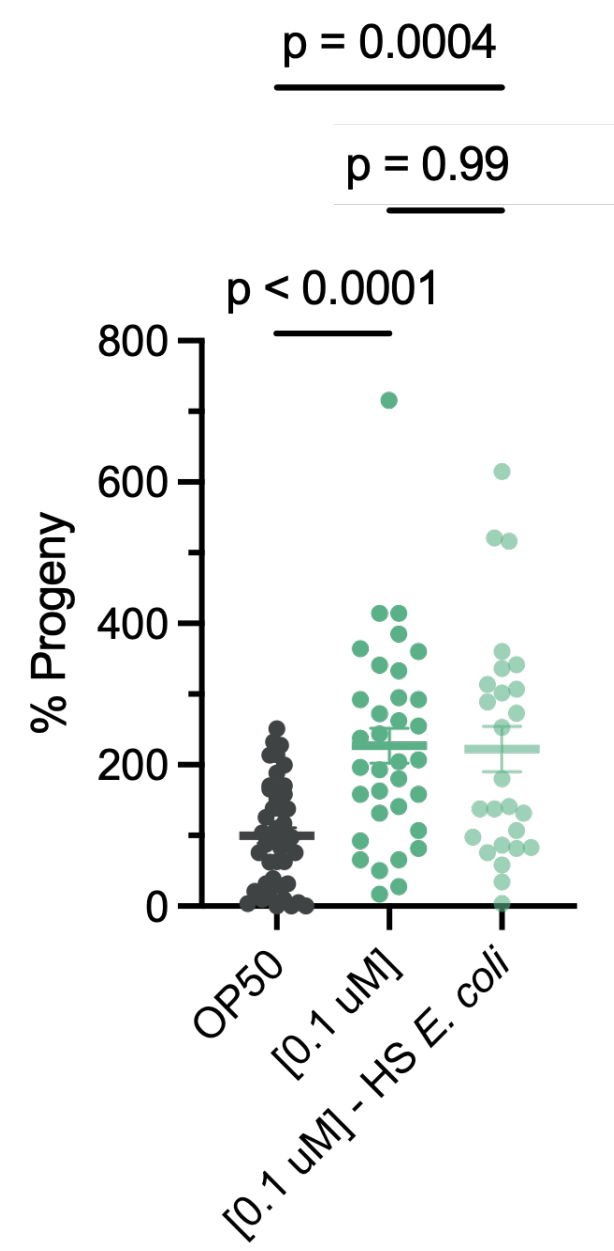

Figure S7. Rescue of the progeny defects in *srdf*-3 W6X worms by atorvastatin is independent of *E. coli.* Heat-shock killed *E. coli* culture does not impact the rescue effect of atorvastatin (0.1 µM) treatment on the progeny phenotype in W6X mutants. n = 44 W6X (OP50), 32 W6X (atorvastatin) and 26 W6X (atorvastatin + heat-shocked bacteria) worms, pooled across 3 experiments. One-way ANOVA with Tukey *post hoc* test. Values are normalized to the mean of the OP50 control. Data are presented as mean ± SEM. Bacterial food source control "OP50". p values are indicated in the figure.

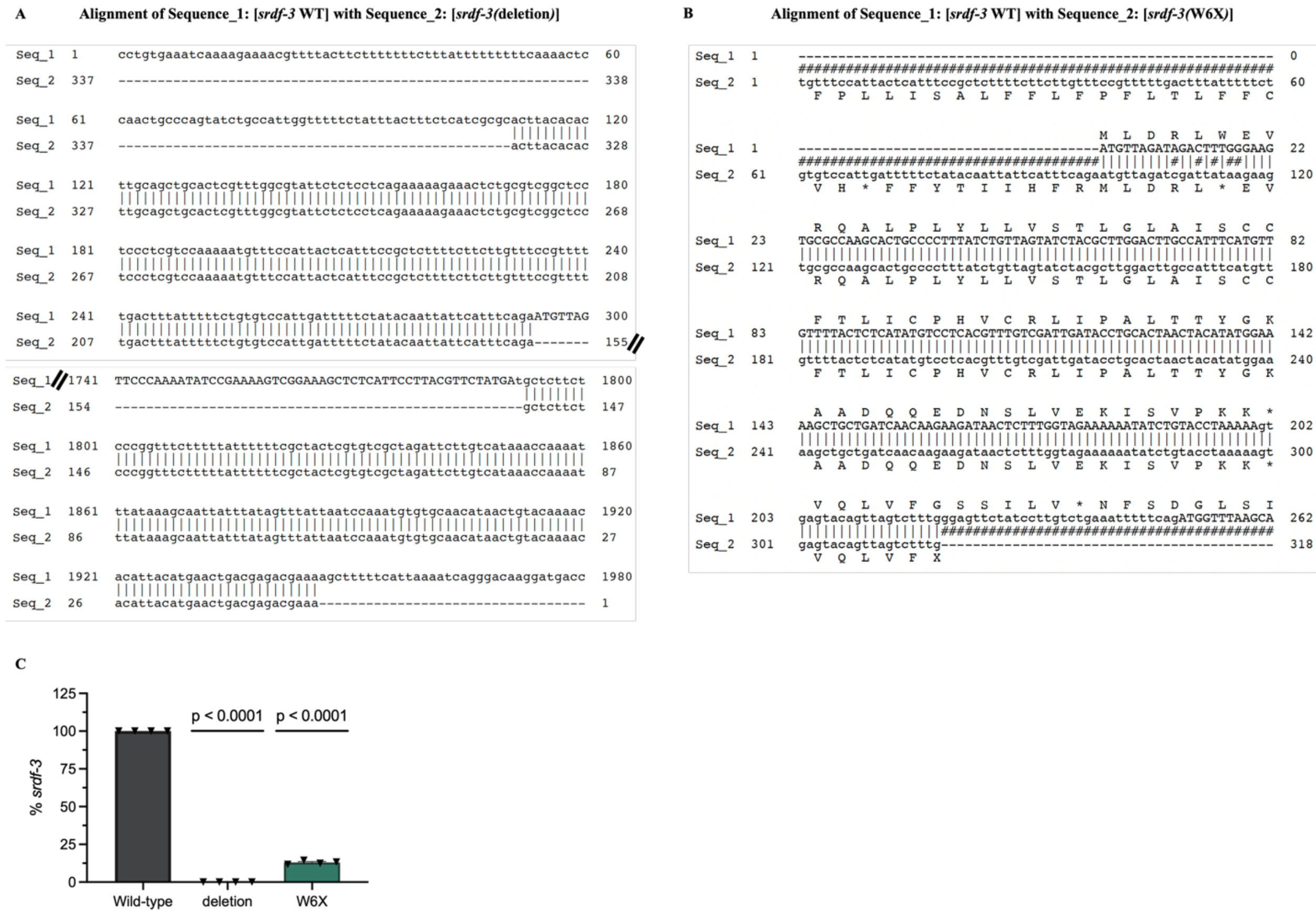

Figure S8. Genotyping of *srdf-3* mutant alleles. (A) Genotyping and sequence alignment of *srdf-3(deletion)* and **(B)** srdf-3(W6X) mutants show the localization of each mutation. **(C)** Quantitative PCR shows a significant decrease in mRNA levels of *srdf-3* in the deletion and W6X mutants. n = 4 independent samples per group. Two-sided one-sample t-test. Data are presented as mean ± SEM. *p* values are indicated in the figure.

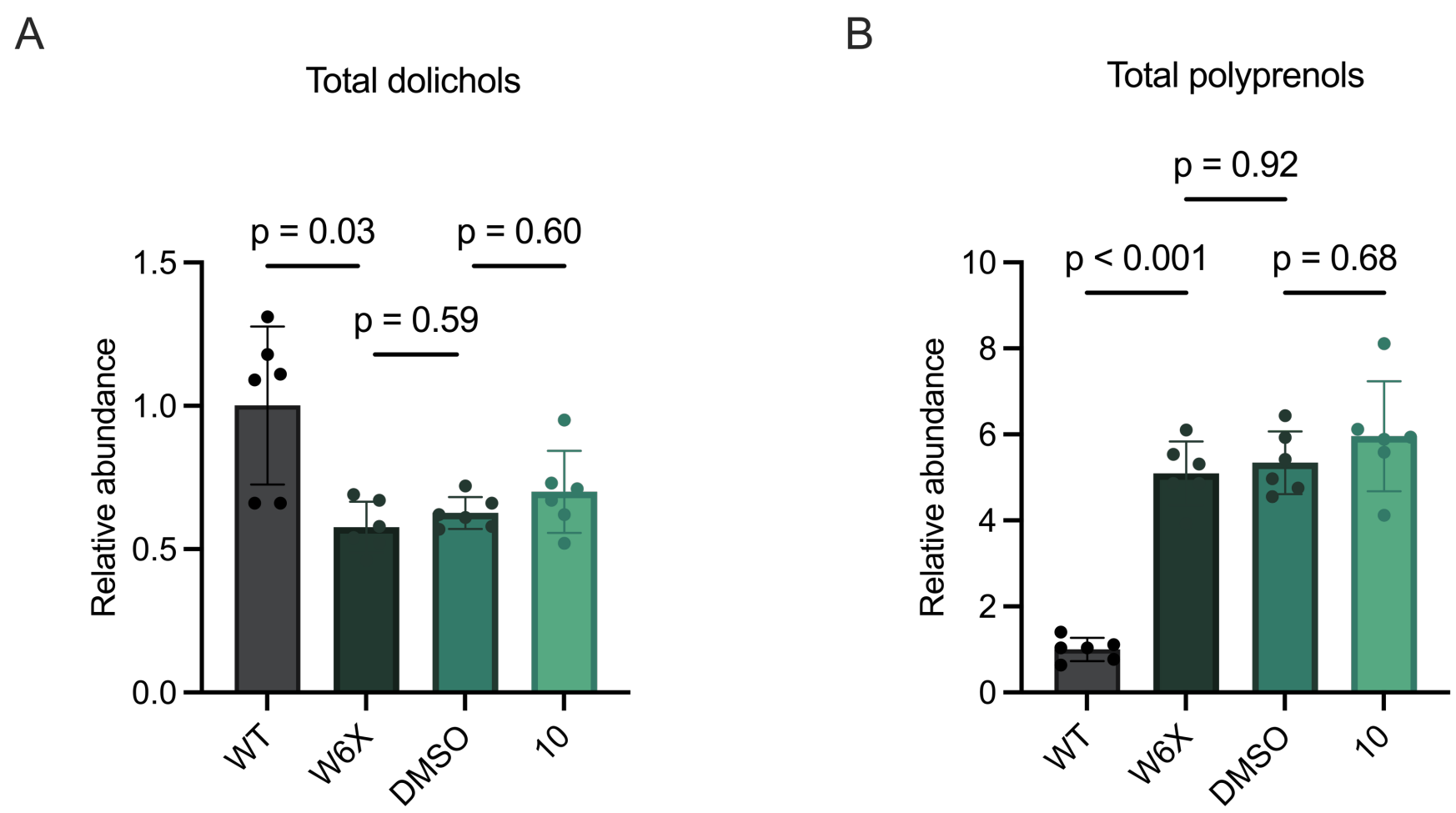

Figure S9. Atorvastatin does not alter dolichol or polyprenol abundance in *srdf-3* W6X worms. Atorvastatin (10µM) does not significantly change **(A)** total dolichols levels or **(B)** total polyprenols levels in W6X mutant worms relative to DMSO-treated control, quantified by LC-MS/MS. *n* = 6 independent samples for WT, DMSO and 10 µM atorvastatin-treated W6X worms. *n* = 3 independent samples for W6X untreated worms. Values are normalized to the mean of WT control. Brown-Forsythe one-way ANOVA with Dunnett *post hoc* test. Data are presented as mean ± SEM. Vehicle-control "DMSO"; wild-type "WT". *p* values are indicated in the figure.

Table S1. Genetic and clinical details of SRD5A3-CDG patients. List of individuals with SRD5A3-CDG used in the study along with genetic information and clinical characteristics.

	**Case 1**	**Case 2**	**Case 3**	**Case 4**
**Age range (y)**	0-5	6-10	6-10	16-20
**Sex**	F	F	M	M
**Predicted protein variant in SRD5A3**	Homozygous large deletion in Exon 5	p.W21X; p.W21X	p.R142X; p.Y163X	p. W19X; p. W19X
**Clinical characteristics**				
Elevation in AST/ALT	+	+	+	+
Abnormal Coagulation	+	+	+	+
Cerebellar hypoplasia	+	+	+	+
Coloboma and visual loss	+	+	+	+
Developmental delay	+	+	+	+
Intellectual disability	+	+	+	+
Ichthyosis	+	-	+	-
Microcytic anemia	+	-	-	-

*Abbreviations: AST – aspartate transaminase; ALT - alanine transaminase

Table S2. *Z*-score and average movement activity across all timepoints of selected compounds identified as positive hits in the secondary screen.

Compound	Z-Score	Activity (% of negative control)
Methiothepin mesylate	2.7856	150.00
Thioridazine hydrochloride	2.2033	130.23
Irigenin trimethyl ether	2.0235	108.86
Pizotifen malate	1.4416	128.97
Perospirone	1.3490	124.86
Zuclopenthixol dihydrochloride	1.2829	127.78
Chlorpromazine hydrochloride	1.2141	124.01
Syringetin-3-glucoside	1.1768	112.35
Cyproheptadine hydrochloride	1.0687	129.84
Carprofen	0.9390	125.20
Hypocrellin A	0.8898	110.36
Loxapine succinate	0.8409	127.16
Piroxicam	0.7533	126.13
Atorvastatin	0.6745	120.62
Hypocrellin B	0.6745	118.90
Chlorprothixene hydrochloride	0.5951	122.62
Isotectorigenin, 7-methyl ether	0.4497	100.59
Ketotifen fumarate	0.4205	122.22
Paliperidone	0.3504	121.40
Loratadine	0.1984	119.64
Acetylsalicylic acid	0.1719	119.44
Dicourmarol	0.1148	104.98

Table S3. ***C. elegans*** **strains and genotypes**

Strain	Genotype	Available from
N2	Wild type *C. elegans* strain	CGC
MOD1	*srdf-3(mod1)*	Parker Lab
MOD2	*srdf-3(mod2)*	Parker Lab
MOD3	*srdf-3*(*mod1*)*;Punc-47p::*mCherry	Parker Lab
MOD4	*srdf-3*(*mod2*)*;Punc-47p::*mCherry	Parker Lab
IZ629	*ufIs34[Punc-47p::*mCherry]	Kind gift from Dr. Michael M. Francis (University of Massachusetts, Worcester, MA)
CB246	*unc-64(e246)*	CGC
CB307	*unc-47(e307)*	CGC
CB1072	*unc-29(e1072)*	CGC
