# Supplementary material for: Repurposing the HMG-CoA reductase inhibitor atorvastatin for SRD5A3-congenital disorder of glycosylation
Source: Commun Med (Lond). 2026 Jul 29;6:517. doi: 10.1038/s43856-026-01791-4 (PMC13642943; doi:10.1038/s43856-026-01791-4)
Supplement: Supplementary file 3 [file 43856_2026_1791_MOESM3_ESM.docx]

**Description of Additional Supplementary Files**

**Supplementary Data 1**: Source data for Figures 1, 2, 3G and Supplementary Figure S8C.

**Supplementary Data 2**: Source data for Figures 3A–F, 5B–C and Supplementary Figures S3 and S9.

**Supplementary Data 3**: Source data for Figures 3I–L, 5F–I and Supplementary Figure S4.

**Supplementary Data 4**: Source data for Figure 4.

**Supplementary Data 5**: Source data for Figures 5A, 5D and 5E.

**Supplementary Data 6**: Source data for Supplementary Figure S5.

**Supplementary Data 7**: Source data for Supplementary Figure S6.
